# Longitudinal Survey on aMPV Circulation in French Broiler Flocks following Different Vaccination Strategies

**DOI:** 10.3390/ani13010057

**Published:** 2022-12-23

**Authors:** Caterina Lupini, Claudia Maria Tucciarone, Giulia Mescolini, Giulia Quaglia, Giulia Graziosi, Vincent Turblin, Paul Brown, Mattia Cecchinato, Matteo Legnardi, Thomas Delquigny, Stéphane Lemiere, Guillaume Perreul, Elena Catelli

**Affiliations:** 1 Department of Veterinary Medical Sciences, University of Bologna, 40064 Ozzano dell’Emilia, BO, Italy; 2 Department of Animal Medicine, Production and Health, University of Padua, 35020 Legnaro, PD, Italy; 3 MC VET Conseil—RESEAU CRISTAL, 72300 Sablé sur Sarthe, France; 4 Laboratoire de Ploufragan-Plouzané-Niort, Agence Nationale de Sécurité Sanitairede l’alimentation, de l’environnement et du Travail, B.P., 53-22440 Ploufragan, France; 5 Boehringer Ingelheim Santé Animale, 69007 Lyon, France

**Keywords:** *avian metapneumovirus*, *infectious bronchitis virus*, broiler, France, longitudinal study, phylogenetic analysis

## Abstract

**Simple Summary:**

In recent years, the impact of respiratory disease resulting from *Avian Metapneumovirus* (aMPV) infection has been rising in the broiler industry in Europe. In this context, in order to investigate aMPV contribution to the clinical picture and the potential benefits of diversified vaccination strategies compared to nonvaccination policies, a longitudinal monitoring was performed in broilers located in Western France. The survey confirmed the circulation of field aMPV subtype B strains with a certain degree of genetic heterogenicity and the importance of implementation of vaccination to contain the clinical manifestations.

**Abstract:**

In recent years, the impact of respiratory disease resulting from *Avian Metapneumovirus* (aMPV) infection has been generally rising in the broiler industry in Europe. In this context, in order to investigate aMPV contribution to the clinical picture and the potential benefits of diversified vaccination strategies compared to nonvaccination policies, a longitudinal monitoring was performed, also evaluating *Infectious Bronchitis Virus* (IBV) presence. Broiler flocks located in Western France, where aMPV has already proven to be a health and productivity issue, were screened by RT-PCR on rhino-pharyngeal swabs, and the viruses were genetically characterized by sequence analysis. For a more comprehensive picture of aMPV molecular epidemiology and evolution in France, aMPV subtype B strains detected from 1985 to 1998 were sequenced and included in the analysis. The survey confirmed the detection of aMPV subtype B in commercial broiler flocks in France, together with a certain heterogeneity demonstrated by the circulation of more recent and historical French field strains. No IBV field strains were detected. The implementation and evaluation of different management choices and vaccine strategies suggests once again that immunization does not prevent infection but contributes greatly to the containment of the clinical manifestations.

## 1. Introduction

*Avian metapneumovirus* (aMPV) is the causative agent of an acute, highly contagious respiratory disease of turkeys, called turkey rhinotracheitis (TRT), and of chickens, that can result in swollen head syndrome (SHS) [1]. Other avian domestic species such as guinea fowl, duck and pheasant can be naturally infected by aMPV [2,3,4,5]. The first appearance of TRT in France (Brittany) dates back to 1981 [6], followed by outbreaks of SHS in chicken and guinea fowl flocks in 1987 [2].

aMPV is an enveloped virus with a negative-sense, single-stranded, unsegmented RNA genome encoding nine proteins: nucleoprotein (N), phosphoprotein (P), matrix (M), fusion (F), matrix 2-1 (M2-1), matrix 2-2 (M2-2), small hydrophobic (SH), attachment (G) and large polymerase (L) [7]. The virus belongs to the order *Mononegavirales*, family *Pneumoviridae*, genus *Metapneumovirus* [8]. When subtyping tests became available at the end of the 1990s, French strains isolated in 1985 and 1986 were assigned to subtypes A, B or D, and those isolated since 1995 were assigned to subtype B [9,10]. The introduction of the B subtype is estimated to have occurred approximatively in 1985 in France, and afterward, the infection spread relatively quickly throughout Europe [11]. Furthermore, aMPV subtype C was identified in France in 1999 from Muscovy ducks exhibiting egg drop after mild respiratory signs [3,12]. During recent diagnostic investigations of respiratory disease outbreaks in poultry, aMPV-B was the sole subtype detected in France; among the affected species, broiler chickens are frequently involved [13], resulting in subsequent direct losses and increased vaccination costs.

Molecular techniques, such as end-point or real-time RT-PCR, are the most widely used tools for the direct detection and, in most cases, the precise subtyping of aMPV strains [9,14,15,16,17]. Owing to its high variability, the G attachment glycoprotein-coding gene represents the major target for molecular differentiation of aMPV-A and aMPV-B vaccine and field strains through sequencing and phylogenetic analysis [13,18] or restriction endonuclease digestion of RT-PCR amplicons [19].

In recent years, the impact of respiratory disease resulting from aMPV infection has been generally rising in the broiler industry in Europe [20,21,22,23], whereas clinical problems related to another important respiratory pathogen, *infectious bronchitis virus* (IBV), appear to be less burdening, likely due to intense vaccination application and the optimization of timing and administration [24]. An interaction between aMPV and IBV has been proposed in different cases [14,25,26], thus being worth investigation. In this context, longitudinal studies were carried out in order to investigate aMPV and IBV circulation and the potential benefits of aMPV diversified vaccination strategies compared to nonvaccination policies. Broiler flocks located in Western France were screened by aMPV and IBV RT-PCRs on rhino-pharyngeal swabs, and portions of the G or S1 genes, respectively, were genetically characterized by sequence analysis. In selected flocks, aMPV had already proven, in previous production cycles, to be a health and productivity issue. aMPV-B strains detected from 1985 to 1998 by the Laboratoire de Ploufragan-Plouzané-Niort of the French Agency for Food, Environmental and Occupational Health & Safety (ANSES) were also sequenced in the same G gene region to assess the evolution of aMPV-B in France and to identify the most effective evidence-based control strategies.

## 2. Materials and Methods

### 2.1. Longitudinal Studies

Broiler flocks and sampling. Between 2017 and 2018, seven broiler flocks (ID numbers: 842, 856, 858, 859, 871, 900, 934) housed in farms located in the Pays de la Loire region in Western France were selected according to their clinical history of SHS. The vaccination program for aMPV is reported in Table 1. Live aMPV subtype B vaccines were administered by spray at the hatchery or at the farm to day-old or 7-day-old chicks. All flocks received infectious bronchitis (IB) vaccination via spray as well (Table 1). Birds (Ross 308) were reared on litter, with dynamic ventilation, and downtime periods ranging from 10 to 34 days were applied.

Rhino-pharyngeal swabs were collected about once a week along the production cycle from twenty randomly selected birds per flock for diagnostic purposes. Dried swabs were stored at room temperature until shipment, and molecular analysis was carried out at the Service of Avian Pathology of Department of Veterinary Medical Sciences, University of Bologna. The sample size was calculated with the aim of detecting at least one positive animal with a confidence level of 99.9%, assuming an animal population size greater than 10,000 chickens and setting the expected minimum prevalence to 30% [27].

The presence of respiratory clinical signs or relevant concurrent health issues was recorded at the sampling time.

Other infectious agents involved in respiratory disease in broilers, such as *Mycoplasmas*, *Newcastle disease* and *influenza A viruses*, were excluded after molecular/serological testing [28,29,30].

### 2.2. aMPV Detection and Sequencing

Ten dried swabs per sampling point for each flock were pooled and immersed in 1 mL of phosphate-buffered saline (Life Technologies Limited, Paisley, UK). RNA was extracted from 140 µL of eluate using QIAamp Viral RNA Mini Kit (QIAGEN, Hilden, Germany) following the manufacturer’s instructions. Samples were processed using a nested RT-PCR targeting the G gene, which enables the simultaneous detection and differentiation of A and B aMPV subtypes [13]. When the assay gave a negative result, the nested RT-PCR was repeated on RNA extracted from the remaining 10 swabs from the same sampling point. The obtained G gene amplicons were purified using ExoSAP-IT™ Express PCR Product Cleanup (Thermo Fisher Scientific, Waltham, MA, USA) according to the manufacturer’s instructions and sequenced in both directions by a commercial sequencing service (Macrogen Europe, Amsterdam, The Netherlands), using the nested RT-PCR primer pair G5- (5′- CAAAGAGCCAATAAGCCCA-3′) and G9+B (5′-TAGTCCTCAAGCAAGTCCTC-3′).

### 2.3. aMPV Sequence and Phylogenetic Analysis

The obtained sequences were assembled using BioEdit Sequence Alignment Editor Version 7.0.5.3 (Tom Hall, Ibis Therapeutics, Carlsbad, CA, USA), aligned and compared, using Clustal W software [31] and Sequence Identity Matrix tool, respectively, to the G gene sequences of aMPV subtype B vaccines (strains VCO3, 11/94, 1062 and PL21), historical B French strains (Table 2) and selected contemporary European B strains [13].

A phylogenetic tree was reconstructed using the maximum likelihood method and Kimura 2-parameter model implemented in MEGA X [32]. The branch support of the phylogenetic tree was assessed by performing 1000 bootstrap replicates, and only branches supported by bootstrap values ≥ 70% were considered reliable.

### 2.4. IBV Detection and Sequencing

Samples were screened for IBV using a nested RT-PCR targeting a hypervariable region of the S1 gene, as described by Worthington et al. (2008) [33], using SuperScript™ III One-Step RT-PCR System with Platinum™ Taq DNA Polymerase kit (Invitrogen, Waltham, MA, USA) for the first amplification and then Platinum™ Hot Start PCR Master Mix kit (Invitrogen, Waltham, MA, USA) for the second PCR. Positive samples were purified and Sanger sequenced as previously described using the primer pair of the second PCR. The obtained sequences were assembled using BioEdit Sequence Alignment Editor Version 7.0.5.3 (Tom Hall, Ibis Therapeutics, Carlsbad, CA, USA), aligned and compared using Clustal W software [31] and Sequence Identity Matrix tool, respectively, to a condensed reference database from Valastro et al. (2016) [34]. A phylogenetic tree was reconstructed using a maximum likelihood method and general time reversible model and gamma distribution implemented in MEGA X [32], performing 1000 bootstrap replicates and considering reliable only branch support >70%.

## 3. Results

**Figure 1:**
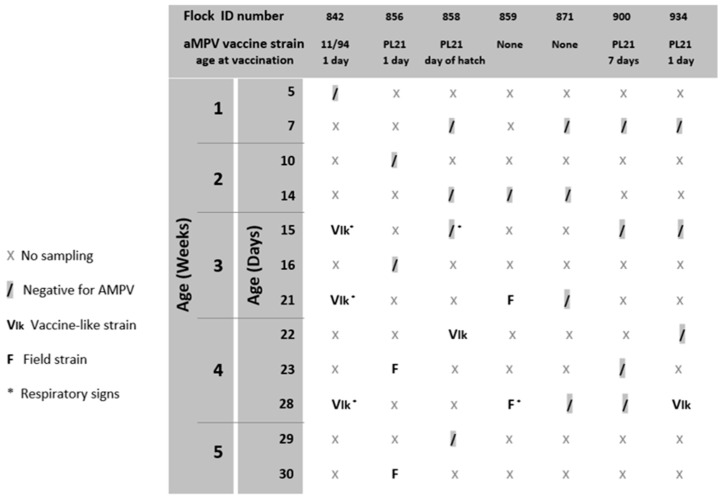
Results of aMPV diagnostic monitoring.

The results of aMPV longitudinal studies are reported in Figure 1. Nine out of forty-nine analyzed swab pools tested positive for aMPV subtype B. Seven out of nine pools were positive after the first extraction; the remaining two tested negatives at first and then positive when the second pool of swabs was analyzed. aMPV G gene sequences and related metadata were deposited in GenBank database (http://www.ncbi.nlm.nih.gov/genbank/ (accessed on 11 October 2022)) under accession numbers OP605527-OP605535. aMPV-B strains were classified as ‘vaccine-like strains’ if the maximum nucleotide sequence identity with a reference vaccine strain was higher than 99% and they fell into the same phylogenetic cluster, or as ‘field strains’ when noncomplying with one or both of the two abovementioned requirements. No IBV field strains were detected.

**Figure 2:**
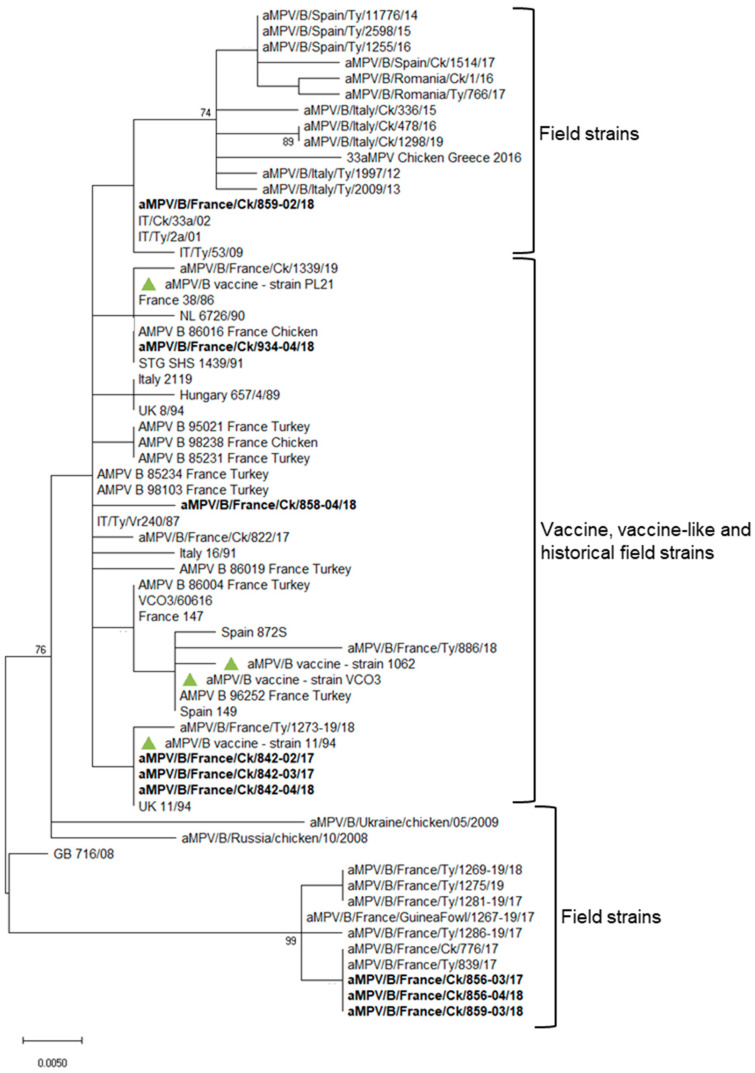
Phylogenetic tree based on G gene nucleotide sequences of French aMPV-B strains. The analysis included B strains detected in broiler longitudinal studies (shown in bold), historical French B field strains, contemporary selected European B strains [13] and B vaccines most commonly used (marked with a green triangle). The evolutionary history was inferred using the maximum likelihood method and Kimura 2-parameter model implemented in MEGA X. All nucleotide positions containing gaps and missing data were eliminated. There were a total of 288 positions in the final dataset. Only bootstrap values ≥ 70% are reported.

**Figure 3:**
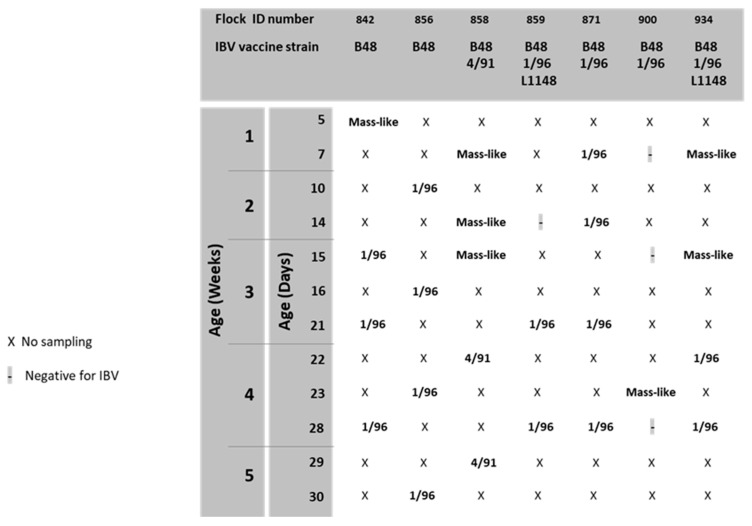
Results of IBV monitoring.

**Figure 4:**
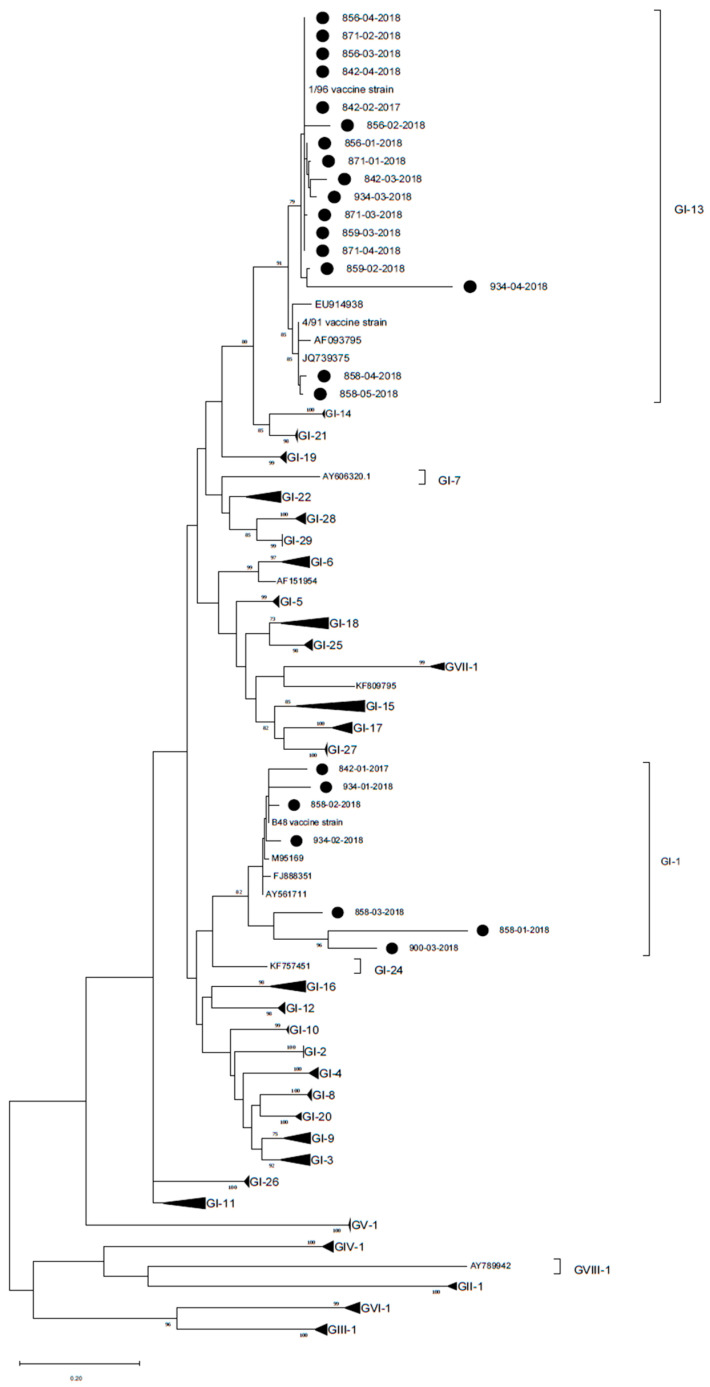
Phylogenetic tree reconstructed using IBV strain references from Valastro et al. (2016) [34]. French strains identified in the present study are marked with a black circle. The tree was reconstructed using the maximum likelihood method and general time reversible model with gamma distribution. Bootstrap support (>70%) is shown next to the branches. The tree is drawn to scale, with branch lengths measured in the number of substitutions per site. This analysis involved 123 nucleotide sequences and a total of 295 positions in the final dataset.

aMPV was detected at 15, 21 and 28 days of age in flock n. 842, where birds were vaccinated at 1 day old at the farm with aMPV-B vaccine strain 11/94 (Figure 1). All detected strains (aMPV-B/France/Ck/842-02/17, aMPV-B/France/Ck/842-03/17 and aMPV-B/France/Ck/842-04/18) were classified as vaccine-like, showing 100% of nucleotide sequence identity and clustering closely with the applied vaccine (Figure 2). Virus detection was associated with respiratory signs. Regarding IBV detection in flock n. 842, a mass-like strain was detected at 5 days of age, accordingly to the vaccination strategy, whereas at the remaining sampling points, 1/96-like strains were identified (Figure 3 and Figure 4).

Identical aMPV field strains (aMPV-B/France/Ck/856-03/17 and aMPV-B/France/Ck/856-04/18) were detected at 23 and 30 days of age in flock n. 856, where birds were vaccinated at 1 day old at the farm with aMPV-B vaccine strain PL21. Phylogenetic analysis showed that the field strains belonged to a cluster of other contemporary field strains (Figure 2). In flock n. 856, overt respiratory signs were absent, and 1/96-like strains were identified at all sampling points, although no vaccines based on this strain were administered, according to the document issued by the hatchery (Figure 3 and Figure 4).

aMPV was detected only at 22 days of age in flock n. 858, whose animals were vaccinated at the hatchery with aMPV-B vaccine strain PL21. At phylogenetic analysis, the detected strain (aMPV-B/France/Ck/858-04/18) fell into a quite heterogeneous cluster containing vaccines, vaccine-like strains and historical field strains. Virus detection followed the appearance of respiratory signs in the flock. No IBV field strains were detected, and the characterized strains reflected the applied vaccination strategy (mass-like strains at day 7 and 14; 4/91-like strains at 22 and 29 days of age) (Figure 3 and Figure 4).

aMPV was detected at 21 and 28 days of age in flock n. 859, unvaccinated against aMPV. The strain aMPV-B/France/Ck/859-02/18 was detected at 21 days of age, clustered with field strains, in particular with Italian ones identified in 2001–2002. At 28 days of age, a field strain (aMPV-B/France/Ck/859-03/18) closely related to recent French field strains was detected concurrently with mild respiratory signs. At the first sampling point, birds were negative for IBV, whereas at 21 and 28 days of age, 1/96-like strains were detected, according to the administered vaccine (Figure 3 and Figure 4).

No aMPV strains were detected during the longitudinal monitoring of flock n. 871, not vaccinated against aMPV, and of flock n. 900, vaccinated at day 7 with aMPV-B vaccine strain PL21. All samples from flock n. 871 were positive for 1/96-like strains according to the vaccination protocol, whereas only one out of four sampling points yielded an IBV-positive sample (mass-like strain at 23 days of age) in flock n. 900 (Figure 3 and Figure 4).

Finally, aMPV was detected at 28 days of age in flock n. 934, vaccinated with aMPV-B vaccine PL21 at 1 day of age. The detected strain (aMPV-B/France/Ck/934-04/18) showed 100% sequence identity with the applied vaccine strain PL21 and belonged to the same phylogenetic group. In this flock, mass-like strains were detected at days 7 and 15, and 1/96-like strains were detected at 22 and 28 days of age, according to IB vaccine strategy.

## 4. Discussion

Since the early 1980s, aMPV has been recognized as one of the major causes of respiratory disorders in poultry, including chickens. In the 1990s, vaccines became commercially available and, since then, have been largely adopted in turkey farming, where aMPV-related disease represents a serious health threat, in contrast to broiler farming where the vaccination is not always considered cost-effective [1]. The benefits of the extension of aMPV vaccination to broiler flocks have been reported in densely populated poultry areas of Northern Italy where the disease is endemic, turkey farms can be found in the vicinity and biosecurity measures are not always strictly applied [21]. Different vaccination strategies and no aMPV immunization have been compared by longitudinal monitoring in seven French broiler flocks, where aMPV-B had already proven in the previous production cycles to be a health issue. aMPV subtype B was the only detected subtype, in accordance with recent epidemiological evidence in several European countries [11,13,20,21,22,23] and in Turkey [35]. Older epidemiological data from other parts of the world reported the detection of subtype A alongside with subtype B, which is usually the dominant subtype [36,37,38]. This trend may be, at least in part, ascribable to an inefficient horizontal transmission of aMPV-A from infected to naïve chickens [39]. The great majority of epidemiological studies on aMPV infection in the chicken focus on subtypes A and B, historically the most relevant subtypes in this species in Europe; therefore, the RT-PCR assay used in the study was designed to detect these subtypes but not C and D subtypes. The only known European subtype C and subtype D isolates, of duck and turkey origin, were isolated in 1999 and 1995 in France [3,9]. Chickens are susceptible to aMPV-C and aMPV-D subtypes in experimental conditions, but they do not efficiently transmit the virus to contact birds [39]. aMPV-C natural infection in chickens has been reported only once, in China [40]. The circulation of these ‘neglected’ subtypes in chicken and turkey flocks should be explored in the future by implementing dedicated molecular diagnostic assays in cases of aMPV suspicion, in the light of the growing report number of aMPV-C detection in minor poultry species and wild birds [41,42,43,44,45,46,47,48], playing a possible epidemiological role. In the present study, vaccination against aMPV was applied to five out of seven broiler flocks, and protocols varied in terms of vaccine strain (strain 11/94 vs. strain PL21) and age of administration (at 1 day old at the hatchery or at the farm or at 7 days of age). Vaccine-like strains with 100% identity with the applied vaccine (strain 11/94) were detected from 15 to 28 days postvaccination, and respiratory signs were reported at the same time in absence of IBV field strains. The prolonged persistence of vaccine strains, observed also for other live aMPV subtype A and B vaccines [49,50], often caused by poor vaccine application, is associated with mild respiratory signs.

In contrast, even if the detection of 1/96 vaccine strains is not rare in farms where the vaccine is not applied, such as in farms n. 842 and 856, the contribution of IB vaccine strains to clinical signs is generally negligible [51]. The presence of IB-modified live vaccines can be often explained by the spread from neighboring flocks, an unintentional contamination during vaccine administration at the hatchery or the persistence from previous cycles [51,52]. These conditions, in addition to the usual replication of administered vaccines, also hamper IB diagnosis, which cannot rely only on generic assays but has to be flanked by strain-specific assays and sequencing for strain characterization [53]. Moreover, different modified live vaccines present peculiar behavior and levels of persistence, likely due to attenuation process, with 793/B-based vaccines replicating longer and at higher titers [26,54], easing the strain persistence and potential spreading among farms [51]. However, the presence of multiple IBV strains with different titers is also difficult to explore with assays yielding a univocal answer, such as PCR amplification and Sanger sequencing [55]; therefore, the general 1/96-like strain detection over mass-like vaccines can be imputed both to the assay choice and vaccine behavior [26,55]. In this context, the late detection of a mass-like vaccine strain only in a farm also vaccinated with 1/96 strain (n. 900) is unusual and could be explained by improper vaccination, suboptimal coverage and a late-stage replication, shedding and circulation of the mass-based vaccine.

However, other infectious agents involved in respiratory disease in broilers, such as Mycoplasmas, were excluded after molecular/serological testing.

Among PL21-vaccinated flocks, a PL21 vaccine-derived strain was detected only in one flock at 28 days of age in the absence of respiratory signs. The lack of PL21 strain detection in the other three PL21-vaccinated flocks could be explained by a short persistence in the upper respiratory tract, coupled with lower replication compared to the widely detected 11/94 strain. Furthermore, IB vaccine strains (mass-like, 4/91 and 1/96 vaccine strains) were identified in these farms. Some authors evidenced that IB vaccines, when applied before aMPV vaccines, can interfere with aMPV vaccine replication, delaying and reducing their detection, although without apparent adverse effect on the induction of protective immunity [25].

In one of the PL21-vaccinated flocks, a different vaccine-like strain clustering with historical French strains (from 1985 and 1998) and vaccines was detected (Figure 2) in presence of respiratory signs. The field origin of this strain cannot be excluded after the acquisition of mutation of the G gene of the currently applied vaccines or of historical strains. The presence of subpopulations in vaccine vials contributing to aMPV heterogeneity and evolution has been also proposed [56]. Sequencing of larger regions of the genome could have offered a clearer picture about the origin of the detected strain.

Field strains sharing their G gene sequences with and belonging to a well-defined phylogenetic clade of contemporary French field strains [13], clearly distinct from historical field strains, were detected in two flocks as early as 23 days of age. One flock was vaccinated with PL21 strain and was asymptomatic; the other was not vaccinated against aMPV, and the birds presented respiratory signs. This clinical evidence could be considered encouraging about the efficacy of the applied vaccination strategy (PL21 strain-based vaccine administration at 1 day of age on the farm) in protecting birds from the clinical disease but not from infection and circulation of field strains, which persisted for at least one week within the flock. Clearly, the outcome of the vaccination in the field and the control of infections can be influenced by multiple environmental and management factors, secondary or concurrent pathogens and biosecurity measures applied. aMPV-B French field strains showed peculiar amino acid changes in the encoded surface G glycoprotein, which contains key epitopes for vaccine-induced immune protection [57]; therefore, the protection conferred by the available vaccines should be reassessed by performing in vivo experimental studies. In fact, the currently adopted live attenuated vaccines were developed in the 1990s by the attenuation of contemporary aMPV field isolates [58,59], which are now reasonably distant from circulating viruses.

In the abovementioned unvaccinated flock (farm n. 859), another aMPV-B field strain clustering with Italian field strains was detected at 21 days of age. Unfortunately, the quality of the obtained partial G gene sequence was quite low, and the short coverage hampered additional analyses. However, the detection of two distinct field aMPV strains in the same flock indicated two separate entry events, suggesting that biosecurity measures should be implemented and a targeted vaccination should be introduced. As a matter of fact, no aMPV strain was detected in two out of seven flocks, of which one was not vaccinated against aMPV and the other one was vaccinated at day 7 with aMPV-B vaccine strain PL21, after clinical signs of swollen head syndrome were noticed in the previous production cycle. This evidence attests to the importance of biosecurity measures, good cleaning and disinfection procedures to prevent field strain entrance in the flocks and persistence between cycles of previously circulating strains.

## 5. Conclusions

The present study confirms the detection of aMPV subtype B in commercial broiler flocks in France, together with a certain heterogeneity demonstrated by the circulation of field strains similar to historical French strains, to Italian strains and to more recent ones. The lack of detection of IBV field strains is in line with the reports of a lower IB prevalence, due to the proper and continuous implementation of vaccination. Regarding aMPV, the evaluation of different management choices and vaccine strategies suggests once again that immunization does not prevent infection but contributes greatly to the containment of the clinical manifestations. However, the use of aMPV-modified live vaccines can confuse the epidemiological picture, as was also reported for IBV, but it can also increase the risk of vaccine reversion to virulence if improperly administered. Therefore, epidemiological monitoring followed by sequencing and strain characterization is pivotal to track and deepen aMPV evolutionary patterns.

## Figures and Tables

**Table 1:** Vaccination programs applied in broiler flocks enrolled in the study.

Flock ID N°	aMPV Vaccine Strain—Age	IBV Vaccine Strain—Age
842	11/94—1 day	B48—day of hatch
856	PL21—1 day	B48—day of hatch
858	PL21—day of hatch	B48—day of hatch 4/91—14 days
859	None—NA	B48 + 1/96—day of hatch L1148—13 days
871	None—NA	B48 + 1/96—1 day
900	PL21—7 days	B48 + 1/96—day of hatch
934	PL21—1 day	B48 + 1/96—day of hatch L1148—14 days

NA—Not applicable.

**Table 2:** Details of historical French aMPV-B strains detected from 1985 to 1998 by the Laboratoire de Ploufragan (ANSES).

Strain Name	Year of Isolation
AMPV B 85231 France Turkey	1985
AMPV B 85234 France Turkey	1985
AMPV B 86004 France Turkey	1986
AMPV B 86016 France Chicken	1986
AMPV B 86019 France Turkey	1986
AMPV B 95021 France Turkey	1995
AMPV B 96252 France Turkey	1996
AMPV B 98103 France Turkey	1998
AMPV B 98238 France Chicken	1998

## Data Availability

The sequences generated in this study are available in GenBank under Accession numbers OP605527-OP605535.
